# Generation of kidney tubular organoids from human pluripotent stem cells

**DOI:** 10.1038/srep38353

**Published:** 2016-12-16

**Authors:** Shintaro Yamaguchi, Ryuji Morizane, Koichiro Homma, Toshiaki Monkawa, Sayuri Suzuki, Shizuka Fujii, Muneaki Koda, Ken Hiratsuka, Maho Yamashita, Tadashi Yoshida, Shu Wakino, Koichi Hayashi, Junichi Sasaki, Shingo Hori, Hiroshi Itoh

**Affiliations:** 1 Department of Internal Medicine, Keio University School of Medicine, 35 Shinanomachi, Shinjuku-ku, Tokyo 160-8582, Japan; 2 Renal Division, Brigham and Women’s Hospital, 75 Francis Street, Boston, MA 02115, USA; 3 Department of Medicine, Harvard Medical School, 25 Shattuck St, Boston, MA 02115, USA; 4 Harvard Stem Cell Institute, 7 Divinity Ave, Cambridge, MA 02138, USA; 5 Emergency and Critical Care Medicine, Keio University School of Medicine, 35 Shinanomachi, Shinjuku-ku, Tokyo 160-8582, Japan; 6 Medical Education Center, Keio University School of Medicine, 35 Shinanomachi, Shinjuku-ku, Tokyo 160-8582, Japan; 7 Apheresis and Dialysis Center, Keio University School of Medicine, 35 Shinanomachi, Shinjuku-ku, Tokyo 160-8582, Japan

## Abstract

Recent advances in stem cell research have resulted in methods to generate kidney organoids from human pluripotent stem cells (hPSCs), which contain cells of multiple lineages including nephron epithelial cells. Methods to purify specific types of cells from differentiated hPSCs, however, have not been established well. For bioengineering, cell transplantation, and disease modeling, it would be useful to establish those methods to obtain pure populations of specific types of kidney cells. Here, we report a simple two-step differentiation protocol to generate kidney tubular organoids from hPSCs with direct purification of KSP (kidney specific protein)-positive cells using anti-KSP antibody. We first differentiated hPSCs into mesoderm cells using a glycogen synthase kinase-3β inhibitor for 3 days, then cultured cells in renal epithelial growth medium to induce KSP+ cells. We purified KSP+ cells using flow cytometry with anti-KSP antibody, which exhibited characteristics of all segments of kidney tubular cells and cultured KSP+ cells in 3D Matrigel, which formed tubular organoids *in vitro*. The formation of tubular organoids by KSP+ cells induced the acquisition of functional kidney tubules. KSP+ cells also allowed for the generation of chimeric kidney cultures in which human cells self-assembled into 3D tubular structures in combination with mouse embryonic kidney cells.

Accumulating evidence revealed critical roles of kidney tubular cells in kidney fibrosis, the most important pathological process which leads to the progress of chronic kidney disease (CKD)^1^,^2^,^3^,^4^,^5^,^6^,^7^,^8^. To facilitate studies on human kidney fibrosis and CKD, it is needed to establish new tools of human kidney tubular cells *in vitro*, since current immortalized human tubular cell lines and primary culture of human proximal tubular cells have limitations due to dedifferentiated phenotypes. Human pluripotent stem cells (hPSCs), by virtue of their unlimited self-renewal and ability to generate cells of all three germ layers of the embryo, are attractive sources for disease modeling as well as regenerative medicine. Significant advances have been made within the past decade that led to generation of kidney lineage cells from mouse and human PSCs^9^,^10^,^11^,^12^,^13^,^14^,^15^,^16^,^17^,^18^,^19^,^20^,^21^,^22^,^23^. Recent studies showed generation of kidney organoids from hPSCs, which contained multiple cell types with characteristics of podocytes, proximal tubules, loops of Henle, and distal tubules^21^,^23^. However, the methods to induce specific cell types of kidneys from hPSCs have not been well-established, which would facilitate studies on kidney bioengineering, cell transplantation, and disease modeling.

Sharmin *et al*. recently demonstrated a method to purify NPHS1-expressing cells from differentiated human induced pluripotent stem cells (hiPSCs), which formed vascularized glomeruli when transplanted into kidney capsules of mice^24^. They generated hiPSCs that express green fluorescent protein (GFP) in the NPHS1 locus. NPHS1 was identified as a causative gene for Finnish-type congenital nephrotic syndrome, and is exclusively expressed in podocytes *in vivo*^25^. Hence, NPHS1-expressing cells are very likely to have characteristics of podocytes, justifying their approach to isolate NPHS1-expressing cells from differentiated hPSCs in order to obtain pure population of podocytes.

To obtain pure population of kidney tubular cells, we focused on one of cadherins, Kidney-specific protein (KSP; cadherin 16) which is exclusively expressed in kidney lineage cells including ureteric buds, developing nephrons, mesonephric tubules, Bowman’s capsules, proximal tubules, loops of Henle, and distal and collecting tubules^26^. We previously generated a monoclonal antibody against the extracellular domain of KSP, which enabled to purify KSP+ cells by flow cytometry^13^. Building on these prior reports, the aim of this study was to establish a rapid and simple differentiation protocol of human embryonic stem cells (hESCs) into kidney tubular cells with purification of KSP+ cells and to generate tubular organoids in 3D culture systems. We employed a feeder-free monolayer culture system to differentiate hESCs into KSP+ cells, and established a simple two-step differentiation protocol using a glycogen synthase kinase-3β (GSK-3β) inhibitor (BIO; 6-bromoindirubin-3′-oxime) in order to obtain sufficient number of KSP+ cells for flow sorting. We also showed that these KSP+ cells derived from hESCs exhibited characteristics of all segments of kidney tubular cell and acquired more functional characteristic when they formed tubular organoids in 3D Matrigel. In addition, we demonstrated that the KSP+ cells form tubular structures in chimeric culture with dissociated mouse metanephric kidneys, supporting our hypothesis that purification of KSP+ cells from differentiated hPSCs provides pure population of kidney tubular cells.

## Results

### Differentiation of hESCs into KSP+ cells

**Figure 1:**
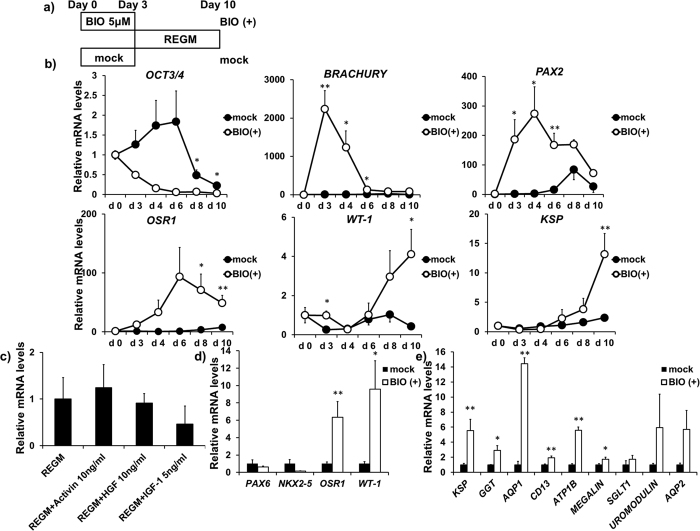
Differentiation of KhES-1 hESCs into a kidney lineage. KhES-1 hESCs formed small clusters of about 200–500 cells on type I collagen-coated dishes following cultivation for 10 days. (**a**) A protocol of differentiation of hESCs sequentially into primitive streak, intermediate mesoderm (IM) and a kidney lineage. In the protocol for primitive streak and intermediate mesoderm (IM), hESCs were differentiated with mock (DMSO) (black circles or bars) or a GSK-3β inhibitor (BIO(+)) (5 μM) (white circles or bars) for 3 days and spontaneously expressed a kidney lineage genes with subsequent stochastic differentiation in Renal Epithelial Growth Medium (REGM) within 10 days of the differentiation beginning. (**b**) Time-course expression of pluripotency (*OCT3/4*) and mesoderm/kidney lineage mesoderm (*BRACHYURY, PAX2, OSR1, WT-1, KSP*) genes in hESCs over 10 days of the differentiation (n = 4–8). (**c**) The effect of activing A, HGF or IGF-1 in *KSP* expression evaluated by real-time PCR on day 10. Activin A, HGF or IGF-1 were added to REGM from day 3 to 10 (n = 2–5). (**d**) Quantitative evaluation of gene expression of ectoderm/endoderm (*PAX6*), cardiac mesoderm (*NKX2–5*), and intermediate mesoderm/kidney lineage (*OSR1, WT-1*) (n = 4–6) and (**e**) kidney tubular (*KSP, GGT, AQP1, CD13, ATP1B, MEGALIN, SGLT1, UROMODULIN, AQP2*) markers on day 10 of differentiation (n = 3–8). Transcript expression levels were normalized to *GAPDH*. Values shown are the means ± SEM. P-values were determined by a Student’s t-test. *P < 0.05; **P < 0.01.

We developed a simple two-step protocol to differentiate KhES-1 hESCs into KSP+ cells within 10 days. Given the facts that embryoid body (EB) formation might be a time-consuming differentiation process and also hinder the effective delivery of differentiation-inducing signals to all cells^27^, we employed a two-dimensional feeder-free monolayer culture system. *In vivo*, the kidneys develop from the intermediate mesoderm (IM) which arises from the primitive streak^28^. Hence, KhES-1 cells were first differentiated into the cells of the primitive streak with a GSK-3β inhibitor (BIO, a WNT activator) (BIO(+); BIO-treated cultures) for 3 days, or without BIO (mock; BIO-untreated cultures) as a negative control (Fig. 1a)^14^,^29^. Accordingly, the expression of the primitive streak marker *BRACHURY*^30^ measured by real time quantitative reverse transcription polymerase chain reaction (qRT-PCR) was transiently increased, whereas the expression of the pluripotency marker *OCT3/4* was down-regulated with BIO treatment (Fig. 1b). Subsequently, those cells were stochastically differentiated with a commercially available renal epithelial growth medium (REGM) for 7 days, which consists of 0.5% fetal bovine serum (FBS), recombinant human epidermal growth factor, insulin, hydrocortisone, epinephrine, triiodothyronine and transferrin^15^,^31^,^32^. We found increased expression of the IM markers, *PAX2* and *OSR1* from day 4 to 6 of differentiation by qRT-PCR (Fig. 1b). The expression of *WT1* was upregulated from day 8 to 10, which is consistent with a previous report showing that *WT-1* is known to be required for kidney development^33^ and restricted to podocytes at later stage^34^ (Fig. 1b). The expression of *KSP*, which is exclusively expressed in kidney lineage cells and occurs during normal development at later stages of kidney tubular differentiation^26^, was significantly greater on day 10 (Fig. 1b). To determine whether additional growth factors to REGM further enhance KSP expression, we tested activin A (10 ng/ml), hepatocyte growth factor (HGF; 10 ng/ml) or insulin-like growth factor 1 (IGF-1; 10 ng/ml) from day 3 to day 10 of differentiation, since our previous study showed that HGF and IGF-1 increased KSP expression in mouse ESCs differentiated with DMEM supplemented with activin A 10 ng/ml^13^. Any of activing A, HGF or IGF-1, however, did not further increase KSP expression compared to REGM alone on day 10 of differentiation (Fig. 1c), suggesting that REGM itself was optimized for differentiation of hESCs into kidney tubular cells. We also evaluated other lineage markers by qRT-PCR in hESCs on day 10 of differentiation; *PAX6* (an endoderm/ectoderm marker) or *NKX2-5* (a cardiac mesoderm marker) (Fig. 1d). Those two genes were not upregulated by our differentiation protocol with BIO and REGM while nephron progenitor markers including *OSR1, WT1, GDNF, CITED1, HOXD11* (Fig. 1d and Fig. S1) and kidney tubular markers including *KSP, GTT, AQP1, CD13, ATP1B, MEGALIN, SGLT1, UROMODULIN, AQP 2* were significantly upregulated (Fig. 1e), suggesting that hESCs were differentiated into kidney lineage cells with the two-step differentiation protocol.

Collectively, these qRT-PCR results indicated that the first specification into the primitive streak cells with BIO for 3 days subsequently induced the intermediate mesoderm cells and kidney lineage cells which represent a heterogeneous population containing cells with characteristics of intermediate mesoderm, metanephric mesenchyme, developing nephron, and glomerular podocytes (*WT1)* and tubular cells (*KSP*) with stochastic differentiation within 10 days of the differentiation beginning.

### Purification of KSP+ cells from differentiated hESCs

**Figure 2:**
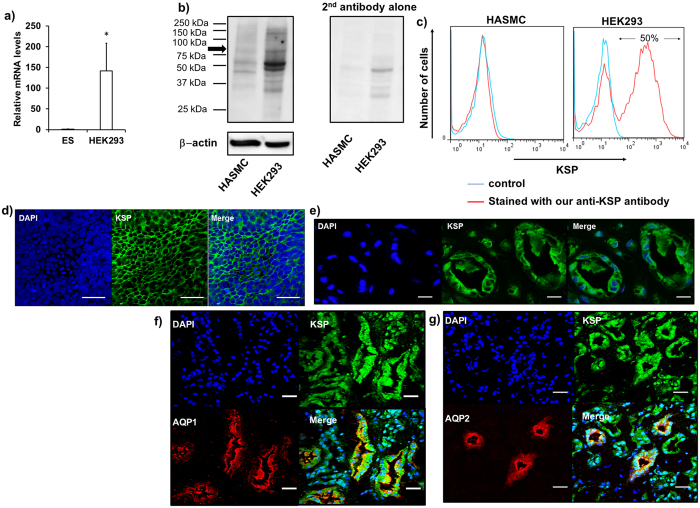
Specificity and cross-reactivity of anti-KSP antibody for human kidney. (**a**) Relative expression levels of *KSP* was determined by real-time PCR for HEK293 cells, relative to the undifferentiated hESCs as a negative control. Transcript expression levels were normalized to *GAPDH* (n = 4–5). Values shown are the means ± SEM. P-values were determined by a Student’s t-test. *P < 0.05 (**b**) Western blot analysis of KSP expression in human aortic smooth muscle cells (HASMCs) and HEK293 cells (left). (right) Immunoblot with secondary antibody alone (2^nd^ antibody alone). (**c**) Flow cytometric analysis of HEK293 cells labeled with our anti-KSP antibody. Blue line represents unstained cells and red represents cells stained with our anti-KSP antibody. HASMCs were used as the negative control. Flow cytometric analysis with our anti-KSP antibody showed positive cells in about 50% of HEK293 cells. (**d**) Immunohistochemistry of HEK293 cells using our anti-KSP antibody. (**e–g**) Human kidney tissue samples were labelled with antibodies against KSP alone (**e**) or in conjunction with AQP1 (**f**) or AQP2 (**g**). Nuclei were counterstained with DAPI. Scale bar; (**d**) 50 μm, (**e–g**) 20 μm.

To obtain pure population of kidney tubular cells from differentiated hESCs with our two-step protocol, we used anti-KSP antibody for flow sorting, which was designed to recognize the extracellular domain of KSP^13^. To evaluate the specificity and cross-reactivity of anti-KSP antibody to human kidney tubular cells, we performed a flow cytometric analysis and immunocytochemistry using a human embryonic kidney (HEK) 293 cell line as a positive control and human aortic smooth muscle cells (HASMCs) as a negative control as indicated by qRT-PCR and western blotting (Fig. 2a,b). Western blotting with anti-KSP antibody detected several bands (Fig. 2b, left). However, a band of 75–100 kDa was not reacted with the secondary antibody alone, suggesting that this band is specific for KSP (Fig. 2b, right). The flow cytometric analysis detected KSP+ cells in HEK293 cells but not in human aortic smooth muscle cells (HASMCs) (Fig. 2c). Immunocytochemistry showed a positive staining on the cell surface of HEK293, which is consistent with protein localization of KSP in kidneys *in vivo* (Fig. 2d)^18^,^35^. In addition, we performed immunohistochemistry of human kidney samples using the anti-KSP antibody, anti-AQP1 antibody, and anti-AQP2 antibody (Fig. 2e–g). KSP+ cells were co-localized with AQP1+ cells which represent proximal tubules (Fig. 2f), AQP2+ cells which represent collecting ducts (Fig. 2g), which is in accordance with the results previously obtained in mouse neonatal kidney tissues and human kidney^13^,^36^. These data demonstrated that our original anti-KSP antibody detected human KSP as well as mouse KSP^13^,^18^.

**Figure 3:**
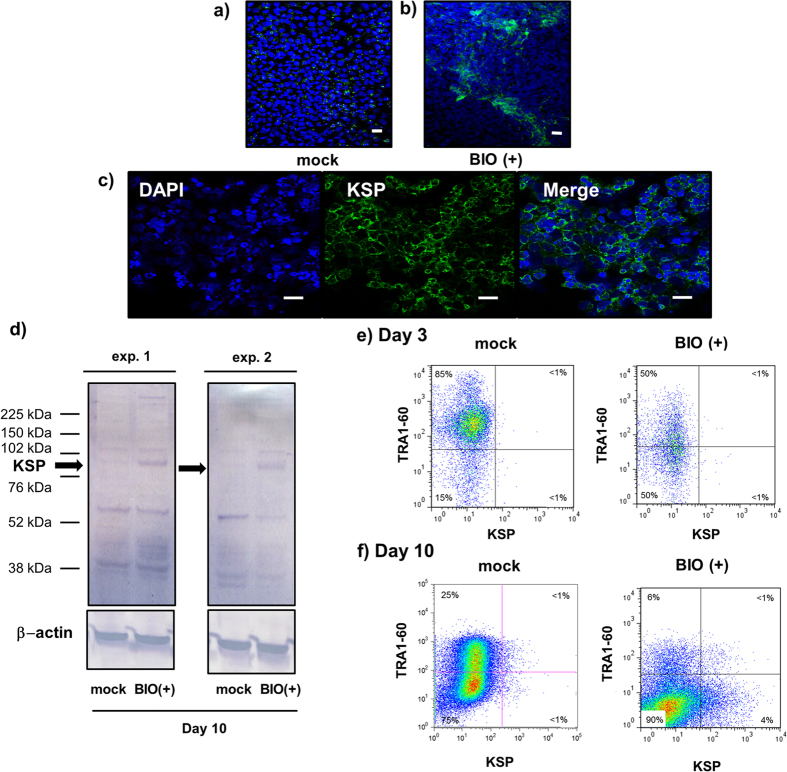
Expression of kidney tubular marker, KSP in differentiating hESCs. (**a–c**) Immunocytochemical analysis of KSP expression in hESCs. HESCs were labelled with an antibody against KSP after 10 d of differentiation in the BIO-untreated (mock) (**a**) or BIO-treated (BIO+) cultures (**b,c**). Nuclei were counterstained with DAPI. Representative images of differentiated hESCs on day 10 of differentiation with a two-step differentiation protocol at low (**b**) and high (**c**) magnification are shown. Scale bar; 20 μm. (**d**) Western blot analysis of KSP expression in hESCs after 10 d of differentiation. KSP expression was not observed in BIO-untreated cells (mock). (**e,f**) Flow cytometric analysis of KSP expression in differentiating hESCs. Expression of TRA1-60 and KSP which are surface markers of hESCs was analysed on day 3 (**e**) and 10 (**f**).

To obtain KSP+ cells from stochastically differentiated hESCs, we evaluated the protein expression of KSP in differentiated hESCs using the anti-KSP antibody. Immunohistochemistry showed that KSP was expressed on the surface of cells in a small population of differentiated hESCs along the periphery of cell clusters on day 10 of the differentiation with BIO and REGM (Fig. 3b,c), whereas KSP staining was not detected in hESCs in BIO-untreated (mock) cells (Fig. 3a), which was consistent with the results obtained by qRT-PCR (Fig. 1b). Western blotting also detected positive bands in samples treated with BIO, but not in samples treated with mock in 2 independent experiments (Fig. 3d).

To exclude the possibility of nonspecific labelling of the anti-KSP antibody, we performed flow cytometry using anti-TRA1-60 (tumour rejection antigen 1-60) antibody in combination with the anti-KSP antibody in hESCs on day 3 and 10 of the differentiation. TRA1-60 is one of pluripotent markers of hESCs and hiPSCs^37^,^38^; therefore, co-expression of TRA1-60 and KSP were very unlikely. On day 3 of the differentiation, KSP+ cells were not detected as predicted by qRT-PCR (Fig. 1b) while TRA1-60+ cells were detected in both mock (85%) and BIO (50%) treated cells (Fig. 3e). On day 10 of the differentiation, KSP+ cells were increased to 4.08 ± 0.514% of differentiated cells with BIO treatment (Fig. 3f), and, KSP and TRA1-60 double positive cells were not detected (<1%). In addition, mock-treated cells were negative for KSP while 25% of cells were positive for TRA1-60 (Fig. 3f). These data are in accordance with the results of qRT-PCR, immunocytochemistry and western blot. (Figures 1b,e and 3a–d). Collectively, these results demonstrated high specificity of our KSP antibody in human samples^18^.

### Characterization of KSP+ cells

**Figure 4:**
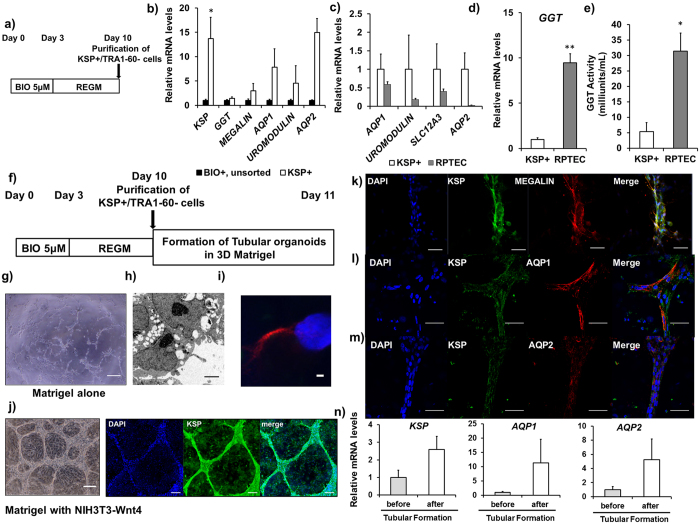
Characterization of KSP+ cells derived from hESCs. (**a**) A protocol to obtain KSP+ cells using flow cytometry from stochastically differentiated hESCs for 7 days after primitive streak induction with BIO for 3 days. (**b,c**) Characterization of KSP+ cells purified from hESCs (KSP+) (white bars) by comparison with unsorted differentiated hESCs on day 10 of differentiation (BIO+, unsorted) (black bars) and primary renal proximal tubular epithelial cells (RPTEC) (gray bars). Relative expression levels of the kidney tubular marker genes indicated were determined by real-time PCR for KSP+ cells, relative to the unsorted differentiated hESCs (**b**) and RPTEC (**c**). (**d,e**) Functional assay of KSP+ cells was determined by GGT activity. (**d**) Relative expression levels of *GGT* were determined by real-time PCR for RPTEC, relative to the KSP+ cells. (**e**) The concentration of p-nitroaniline (pNA) produced by KSP+ cells and RPTEC was measured and normalized to the cell number. (n = 3). (**f–k**) A protocol to form tubular organoids by KSP+ cells in 3D Matrigel. KSP+/TRA1-60 negative cells purified by flow cytometry after 10 d of differentiation were transferred onto Matrigel and cultured for 24–48 h. Light microscopy (**g**), electron microscopy of a cross section (**h**) and immunostaining for acetylated tubulin (**i**) of tubular organoids formed by KSP+ cells. (**j–m**) After 24 h, KSP+ cells co-cultured with NIH3T3-Wnt4 formed tubular organoids that contained MEGALIN (**k**), AQP1 (**l**), or AQP2 (**m**) positive cells. After 48 h, the relative expression levels of the kidney tubular marker genes indicated were determined by real-time PCR for KSP-positive cells before and after tubular formation (**n**). Scale bar; 500 μm (**g**), 2 μm (**h,i**), (**j**) 500 μm (left), 100 μm (right), 50 μm (**k**–**m**). Transcript expression levels were normalized to *GAPDH* (n = 2–6). Values shown are the means ± SEM. P-values were determined by a Student’s t-test. *P < 0.05; **P < 0.01.

We confirmed the presence of sufficient KSP+ cells in hESCs by flow cytometry on day 10 of the differentiation with our two-step protocol (Fig. 3f). To characterize KSP+ cells purified from differentiated hESCs with our two-step differentiation protocol (Fig. 4a), we purified KSP+ cells from differentiated hESCs with flow cytometry using the KSP-antibody on day 10 of the differentiation and evaluated gene expression of kidney tubular markers by qRT-PCR. Gene expression in KSP+ cells were compared to that in whole differentiated hESCs on day 10 of the differentiation (BIO+, unsorted, Fig. 4b), which were not subjected to flow sorting, in order to evaluate how efficiently flow sorting with the KSP-antibody improved purity of kidney tubular cells. KSP+ cells purified by flow sorting showed significantly higher expression of *KSP* than unsorted hESCs by qRT-PCR (Fig. 4b), demonstrating that flow sorting with the KSP-antibody significantly improved purity of KSP+ tubular cells. qRT-PCR also showed that *MEGALIN, AQP1, UROMODULIN* and *AQP2* were higher in KSP+ cells than in unsorted hESCs (Fig. 4b).

In order to further characterize purified KSP+ cells, we compared gene expressions of kidney tubular markers in KSP+ cells to those in primary renal proximal tubular epithelial cells (RPTECs). KSP+ cells showed a comparable expression of *AQP1* as RPTECs, whereas the expressions of *UROMODULIN, SLC12A3* and *AQP2* were higher in KSP+ cells than RPTECs (Fig. 4c). These data suggest that KSP+ cells have characteristics of proximal tubular, loops of Henle, distal tubular and collecting duct cells^39^,^40^,^41^,^42^.

We then evaluated the functional characteristics of purified KSP+ cells by mRNA expression and activity of γ-glutamyl transferase (GGT), which is known to be expressed in the proximal tubule^15^,^43^. The modest increase of *GGT* in purified KSP+ cells compared with unsorted hESCs on day 10 of the differentiation (1.45 ± 0.25-fold) is most likely due to the fact that the basal level of *GGT* mRNA level in unsorted hESCs are already high (Fig. 4b), since GGT is expressed in various tissues^43^. In order to analyse the GGT activity, which reflects the functional characteristic of proximal tubular cells^15^, we measured p-nitroaniline which is released by the action of GGT^15^. KSP+ cells as well as RPTECs demonstrated GGT activity (Fig. 4e); however, the GGT activity in the RPTECs was about six times as high as that in KSP+ cells, consistent with the relative gene expression level of *GGT* in KSP+ cells and RPTECs (Fig. 4d). These results suggest that KSP+ cells are a heterogeneous population containing not only proximal tubules but also other segment cells, though another possible explanation is that KSP+ cells are functionally less mature than RPTECs. In addition, as our previous study showed that tubular formation in Matrigel with Wnt4 further promoted differentiation of KSP+ cells derived from mESCs into more functional proximal tubules and collecting duct cells^13^, we hypothesized that same approaches would further facilitate functional maturation of KSP+ cells derived from hESCs. First, we investigated the capacity of KSP+ cells for tubular organoids formation *in vitro* by transferring onto Matrigel (Fig. 4f). KSP+ cells formed small and sparse tubular structures in Matrigel alone (Fig. 4g). Cross-section slides of those tubular structures showed cilia-like structures at the luminal surface (Fig. 4h). Consistent with this, staining of the ciliary marker, acetylated TUBULIN was observed^18^, indicating that kidney tubular organoids generated from purified KSP+ cells have cellular polarity (Fig. 4i). Furthermore, we tested a co-culture with NIH3T3-Wnt4 cells that constitutively express Wnt4, which is essential for tubulogenesis^13^,^44^. After 24 h of culture, the KSP+ cells formed abundant tubular organoids that co-expressed MEGALIN, AQP1 or AQP2 (Fig. 4j–m), demonstrating that KSP+ cells have characteristics of kidney tubular cells. In contrast, KSP negative cells just formed petal-like structures that were negative for KSP and MEGALIN (Fig. S2). Accordingly, the gene expression of *KSP, AQP1*, and *AQP2* was increased upon Wnt4 stimulation after 48 h of co-culture (Fig. 4n), suggesting the induction and maturation of proximal tubules and collecting ducts. These findings indicate that KSP+ cells possess the functional characteristics of kidney tubular cells and formed tubular organodis in 3D Matrigel.

### Formation of tubular structures in chimeric culture with dissociated mouse metanephric kidneys

**Figure 5:**
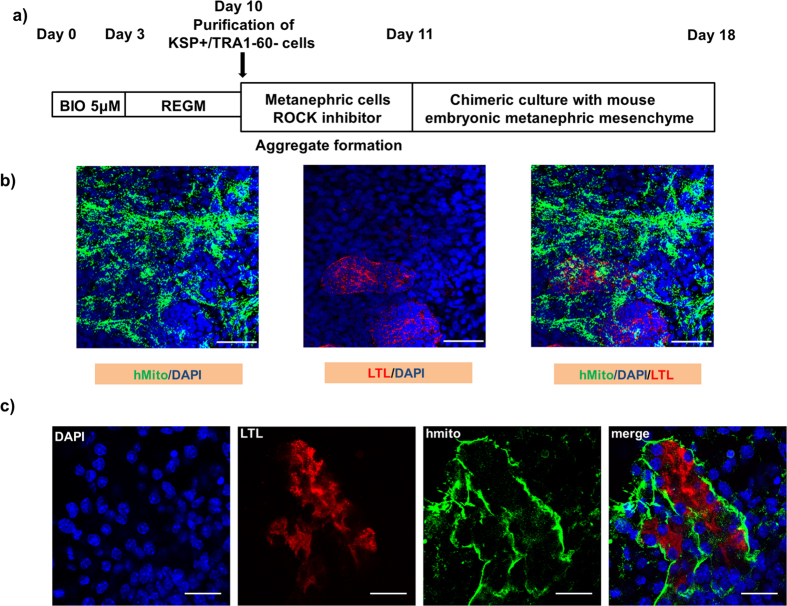
KSP+ cells form tubular structures in chimeric culture with dissociated mouse metanephric kidneys. (**a**) A protocol of chimeric culture with dissociated mouse metanephric kidneys. KSP+/TRA1-60 negative cells purified by flow cytometry and freshly dissociated metanephric cells were incubated with ROCK inhibitor overnight and formed aggregates were incubated for 1 week. Sections of resulting aggregates were examined by immunostaining and imaged by microscopy. Samples were labelled with antibodies against human mitochondria (Mito) in conjunction with LTL (**b,c**). Scale bars; (**b**) 50 μm, (**c**) 20 μm.

To confirm our results that KSP+ cells have characteristics of kidney tubular cells, we examined functional integration of KSP+ cells into 3D renal structures during the re-aggregation of embryonic mouse metanephric cells, as described previously^14^,^17^,^27^,^45^. In this assay, dissociated cells from wild-type E11.5 mouse embryonic kidneys, which can recapitulate nephrogenesis^27^,^45^ were re-aggregated with KSP+ cells purified from differentiated hESCs on day 10 of the differentiation (Fig. 5a). Since a previous study demonstrated that undifferentiated hPSCs disrupted the formation of 3D structures by murine cells^27^, we excluded undifferentiated hESCs with anti-TRA1-60 antibody. After 7 days of chimeric culture in REGM, we performed immunohistochemistry for human mitochondria in order to identify hESC-derived cells (Fig. 5b,c). Human mitochondria positive cells formed 3D tubular structures positive for the proximal tubule marker, lotus tetragonolobus lectin (LTL) (Fig. 5b,c) in combination with mouse embryonic kidney cells. These data demonstrate that KSP+ cells are committed to kidney tubular cells.

## Discussion

We demonstrated a rapid and simple differentiation protocol of hESCs into kidney tubular cells, direct purification of kidney tubular like cells with the anti-KSP antibody and generation of tubular organoids in 3D culture systems. We have previously shown that mESC-derived KSP+ cells exhibit characteristics of kidney tubular phenotypes^12^,^13^ and have suggested that KSP can serve as a kidney lineage marker during mESC differentiation. Here, we successfully generated the KSP+ cell population from hESCs with stochastic differentiation after induction of primitive streak in approximately half the time compared to that required for the differentiation of mouse ES cells^12^,^13^. In addition, KSP+ cells derived from hESCs exhibited the functional characteristics of kidney tubular cells, formation of tubular organoids in 3D Matrigel, and the generation of chimeric kidney cultures.

In the present study, the differentiation of hESCs was achieved by a monolayer culture method that did not require feeders, high serum concentration, or EB formation. Previous studies have reported that the signalling induced by extracellular matrix components plays an important role in directing early kidney differentiation, suggesting that this process might be promoted in a monolayer culture^46^. On the other hand, EB formation and feeder cell contamination might hinder the initiation of such signalling^14^,^27^, and certain growth factors present in serum have been shown to antagonize hESC differentiation^47^.

The inhibition of GSK-3β, which is a serine/threonine kinase, leads to the activation of canonical Wnt/β-catenin signalling in ESCs, resulting in mesodermal differentiation^14^,^45^,^48^,^49^,^50^,^51^,^52^. Therefore, during the first step of our protocol, KhES-1 cells were first differentiated into the cells of primitive streak cells with a GSK-3β inhibitor (BIO). As shown in this study, treatment of BIO induced *BRACHYURY* expression (Fig. 1b), indicating the induction of the primitive streak cells. Notably, previous studies suggested that the *Brachyury*-positive primitive streak population is capable of differentiating into the *Osr1*-positive IM cells, the origin of kidney lineage^16^,^28^. The induction of primitive streak cells from hPSCs is triggered by the activation of Wnt/β-catenin signalling, and subsequent differentiation into IM cells requires downregulation of Wnt activity^16^,^49^. Therefore, in the second step of our protocol, we removed the Wnt/β-catenin stimulation and provided culture conditions suitable for the efficient generation of IM cells and kidney tubular cells^15^,^31^,^32^. In our step-wise procedure, the treatment of KhES-1 cells with BIO for 3 d induced *Brachyury* expression resulting in a decrease of TRA1-60+ cells (Figs 1b and 3e), and subsequent culture in REGM induced the differentiation into KSP+ cells (Figs 1b and 3f). In contrast, BIO-untreated cultures did not induce KSP+/TRA1-60-negative cells on day 10 of differentiation (Fig. 3f). These results demonstrated that application of the GSK-3β inhibitor in conjunction with culture in REGM can efficiently promote the differentiation of hESCs into KSP+ kidney tubular cells.

Under these differentiation conditions, the gene expression of not only *KSP* but also *WT1*, which is known to be expressed in intermediate mesoderm, metanephric mesenchyme, developing nephron, and glomerular podocytes at the late stage of kidney differentiation^34^, was upregulated on day 8~10 of differentiation. Given that the cell population after differentiation was heterogeneous, the KSP+/TRA1-60-negative cells were purified from differentiated hESCs using flow cytometry (Fig. 3f) to obtain pure population of kidney tubular cells.

Next, we evaluated the functional characteristics of purified KSP+ cells. *GGT* is expressed in the proximal tubule^15^,^43^ and produces p-nitroaniline. Thus, GGT activity reflects the functional characteristic of kidney tubular cells^15^ and KSP+ cells demonstrated GGT activity (Fig. 4e). KSP+ cells also formed tubular organoids that contained MEGALIN+, AQP1+ or AQP2+ tubular cells in 3D Matrigel (Fig. 4j–m). By forming tubular organoids, KSP+ cells acquired more functional characteristic with higher expression of functional tubular proteins including AQP1 and AQP2 (Fig. 4n), which represent proximal tubules and collecting ducts respectively. Previously, Narayanan *et al*. and Kandasamy *et al*. showed the induction of proximal tubule-like cells with the high purity, by differentiation of hPSCs with REGM supplemented with bone morphogenetic protein (BMP) 2 and 7 and formed tubule-like structures in a two-dimensional culture^15^,^53^. In contrast to their studies, here we were able to purify KSP+ cells that exhibited characteristics of proximal tubular, loops of Henle, distal tubular and collecting duct cells, which is consistent with previous studies showing KSP expression in all segment of tubules from proximal tubule to collecting ducts^26^. KSP+ cells also generated tubular organoids that contained MEGALIN+, AQP1+ or AQP2+ tubular cells from KSP+ cells in 3D Matrigel.

In addition, KSP+/TRA1-60-negative cells formed tubular structures in chimeric kidney cultures with dissociated mouse metanephric kidneys in organ culture experiments, indicating that KSP+ cells purified from differentiated hESCs provides a population of definitive kidney tubular lineage cells.

## Conclusions

In this study, we have demonstrated a method to purify human kidney tubular cells from hESCs with the anti-KSP antibody after differentiation with a simple two-step differentiation protocol. This approach is useful for generating cells of definitive kidney tubular lineage cells that might be usable as a source for generation of bioengineered kidneys, modeling kidney diseases, drug screening for new therapeutic approaches for kidney diseases, nephrotoxicity assays, and ultimately kidney regenerative medicine using hPSCs.

## Materials and Methods

### Undifferentiated human ES cell culture

KhES-1 (46, XX) human ES cells (kindly provided by Kyoto University) were grown on mouse embryonic fibroblast (MEF) feeder layers (derived from embryonic day (E) 14.5 ICR mouse embryos and subsequently treated with mitomycin C (Sigma-Aldrich, St. Louis, MO, USA) and maintained at 37 °C and 5% CO_2_. KhES-1 and MEFs were co-cultured in ES cell maintenance medium containing 77% Dulbecco’s modified Eagle’s medium (DMEM)/F12, 20% knockout serum replacement (Invitrogen, Carlsbad, CA, USA), 1% nonessential amino acids by volume (Invitrogen), 2 mM L-glutamine (Sigma-Aldrich), and 0.1 mM 2-mercaptoethanol (Gibco, Carlsbad, CA, USA), supplemented with 4 ng/ml recombinant human basic fibroblast growth factor (Wako Pure Chemical Industries, Ltd., Osaka, Japan). For routine passaging, every 5 or 6 days undifferentiated KhES-1 cells were detached with dissecting pipettes (Costar, Washington, DC, USA) and split at a ratio between 1:3 and 1:6.

### Human ES cell differentiation

Undifferentiated KhES-1 cell colonies were detached with a solution containing 0.25% trypsin (Invitrogen), 20% knockout serum replacement (Invitrogen), 1 mg/ml collagenase (Wako) by volume, and 1 mM CaCl_2_ (Wako) in phosphate-buffered saline (PBS) (Gibco), and dissociated into small cell clusters. Prior to differentiation, the cell clusters were filtered through a 40-μm nylon mesh cell strainer (BD Biosciences, San Jose, CA, USA) to remove MEFs.

To induce KhES-1 cell differentiation, cell clusters were transferred to type I collagen-coated dishes (Asahi Techno Glass Co., Ltd., Tokyo, Japan) and cultured for 1 day in DMEM/F12, 20% knockout serum replacement, 1% nonessential amino acids by volume, 2 mM L-glutamine, and 0.1 mM 2-mercaptoethanol, which was then replaced with DMEM/F12, 20% knockout serum replacement, 1% nonessential amino acids by volume, 2 mM L-glutamine, and 0.1 mM 2-mercaptoethanol supplemented with B27, N2 (Invitrogen), and 5 μM GSK-3β inhibitor (BIO; Sigma-Aldrich) for 3 days (days 0–3). The medium was then changed to REGM supplemented with REGM SingleQuots (Lonza, Basel, Switzerland) containing 0.5% fetal bovine serum (FBS), and 0.1% recombinant human epidermal growth factor, insulin, hydrocortisone, epinephrine, triiodothyronine, transferrin, and gentamycin by volume. Cells were then incubated for an additional 7 days (days 3–10).

### Flow cytometry

Cells were dissociated with trypsin/EDTA (Invitrogen) and treated with 0.2% collagenase type IV (Wako) for 15 min at 37 °C. The cells were mechanically dissociated by pipetting, then filtered through a 70-μm cell strainer (Falcon, Corning, NY, USA) and maintained in DMEM containing 10% FBS for 1 h before treatment with 10 μg/ml Fc blocker (#422301; Bio Legend, San Diego, CA, USA) for 20 min on ice. The cells were incubated with 0.08 mg/ml biotinylated anti-KSP antibody (originally produced using hybridomas)^13^ for 30 min, 0.01 mg/ml streptavidin-conjugated Alexa Fluor 647 (Invitrogen) for 20 min on ice, and finally with a fluorescein isothiocyanate (FITC)-conjugated monoclonal anti-TRA1–60 antibody (#560173; 1:200; BD Biosciences). Just prior to analysis, the cells were transferred to a 5 ml polypropylene tube (Falcon, Corning) to which 1 μg/ml propidium iodide (Sigma-Aldrich) was added. Cells were counted and sorted using a flow cytometer (MoFlo XDP; Beckman Coulter, Brea, CA, USA) according to the manufacturer’s instructions. The KSP expression threshold was set relative to the signal intensity of negative control samples incubated without antibody or with the secondary antibody only.

### Tubular organoid formation in 3D Matrigel

NIH3T3 cells that constitutively express Wnt4 (NIH3T3-Wnt4; kindly provided by Dr. Andy McMahon, University of Southern California, USA) were maintained in DMEM supplemented with 10% FBS. Cells were expanded in culture dishes after being mitotically inactivated by 10 μg/ml mitomycin C for 2 h 45 min. Matrigel (BD Biosciences) was placed on inactivated NIH3T3-Wnt4. KSP-positive/TRA1-60 negative cells purified from KhES-1 cells were transferred onto Matrigel (BD Biosciences) in REGM and cultured for 24–48 h.

### RNA extraction, cDNA synthesis, PCR, and real-time PCR

Total RNA was isolated using an miRNeasy Mini Kit (Qiagen, Valencia, CA, USA) and converted to cDNA using the High-Capacity Reverse Transcription Kit (Applied Biosystems, Carlsbad, CA, USA) according to the manufacturer’s instructions. Real-time PCR was performed using a QuantiFast SYBR Green PCR Kit (Qiagen) according to the manufacturer’s protocol. Expression levels were calculated using the 2−ΔΔCt method and normalized to levels of the internal control glyceraldehyde-3-phosphate dehydrogenase (*GAPDH*).

### Immunolabelling

Human kidney tissue samples from two donors (OTB-2141; paraffin embedded kidney sections from a 62 years old, male; Capital Biosciences, Rockville, MD, USA, and HuFTS241; frozen kidney sections from a 54 years old, male; Biomax US Inc. Rockville, MD, USA) were immunolabelled as follows. Formalin-fixed paraffin embedded tissues were deparaffinised by two changes each of xylene and 100% ethanol, and then hydrated, followed by immersion in a target retrieval solution (Dako, Carpinteria, CA, USA). Frozen tissue sections were fixed in 4% paraformaldehyde (PFA) (Wako) in PBS for 15 min at room temperature. Both slides were blocked in PBS containing 4.0% bovine serum albumin (BSA) (Nichirei Biosciences, Inc., Tokyo, Japan) and 0.1% Triton X-100 (Wako), and incubated at 4 °C overnight with one of the following primary antibodies diluted in 0.1% BSA/PBS: anti-KSP (1:100; originally produced by hybridomas), anti-AQP1 (#SC20810; 1:50; Santa Cruz Biotechnology, Inc., Dallas, TX, USA), or anti-AQP2 (#A7310; 1:200; Sigma-Aldrich). After washing with PBS/0.1% Tween 20 (Wako), the sections were incubated with Alexa Fluor 488- or 594-conjugated secondary antibodies (1:500; Invitrogen) for 1 h at room temperature. After counter-staining with DAPI (300 nM in PBS; Invitrogen) to visualize the nuclei, images were obtained using a LSM710 confocal microscope (Carl Zeiss Microscopy GmbH, Göttingen, Germany).

KhES-1 cells on day 10, incubated with or without BIO (5 μM) for the first 3 days of differentiation and HEK 293 cell line were stained after fixation with 4% PFA in PBS. Samples were blocked with PBS containing 4% BSA and incubated with anti-KSP antibody (1:100; originally produced by hybridomas), followed by an Alexa Fluor 488-conjugated secondary antibody (1:200; Invitrogen).

KSP-positive/TRA1-60 negative cells cultured in Matrigel with or without NIH3T3-Wnt4 cells and chimeric culture with dissociated mouse metanephric kidneys were blocked with PBS containing 4.0% BSA and 0.1% Triton X-100, then incubated with one of the following primary antibodies: anti-KSP (1:100; originally produced by hybridomas)^13^,^18^, anti-human-specific mitochondria (#ab3298; 1:50; Abcam), anti-AQP1 (#SC20810; 1:50; Santa Cruz Biotechnology), anti-AQP2 (#A7310; 1:200; Sigma-Aldrich), anti-Acetylated Tubulin (#T6793; 1:500; Sigma-Aldrich) or FITC-conjugated lotus tetragonolobus lectin (#FL-1321; LTL, 1:200; Vector Laboratories, Burlingame, CA, USA). After PBS washes, Alexa Fluor 488- or 594-conjugated antibodies (Invitrogen) and streptavidin-conjugated Alexa Fluor 647 (Invitrogen) were applied for signal detection.

### Western blot analysis

Cells were washed twice with PBS and lysed in radioimmunoprecipitation buffer composed of 20 mM Tris-HCl (pH 7.4), 0.1% sodium dodecyl sulphate (SDS), 1% Triton X-100, and 1% Na deoxycholate (all from Wako). Protein lysates (20 μg) were resolved by 10% Tris-glycine SDS polyacrylamide gel (Bio-Rad Laboratories, Inc., Hercules, CA, USA) electrophoresis using an Any kD precast gel (Bio Rad Laboratories) and transferred to Immobilon polyvinylidene fluoride membranes (Millipore, Marlborough, MA, USA), which were blocked with Tris-buffered saline containing 5% dried milk (Megmilk Snow Brand Co., Ltd., Hokkaido, Japan). Western-blotted protein extracts from KhES-1 cells were probed with antibodies against KSP (originally produced by hybridomas^13^,^18^ or #ab116368; Abcam, Cambridge, MA) and β-actin (#A1978; Sigma-Aldrich) (loading control). The membranes were then incubated with alkaline phosphatase-conjugated anti-mouse or anti-rabbit secondary antibody (Promega, Madison, WI, USA), and horseradish peroxidase (HRP)-conjugated anti-mouse antibody (Jackson ImmunoResearch Laboratories, West Grove, PA). Signals were detected using an alkaline phosphatase (Promega) or ECL detection system (Amersham).

### Chimeric kidney cultures

Metanephric kidneys from imprinting control region (ICR) mice (Japan SLC, Inc., Shizuoka Prefecture, Japan) at embryonic day 11.5 were dissected in improved MEM (Invitrogen). Metanephric cells were dissociated in 0.05% trypsin/EDTA (Sigma-Aldrich) in PBS for 10 min at 37 °C, stabilized in improved MEM containing 1% penicillin/streptomycin (Sigma-Aldrich) and 10% FBS for 10 min, then filtered through a 40-μm cell strainer. A total of 1 × 10^5^ freshly dissociated metanephric cells and 1 × 10^4^ KSP-positive/TRA1-60 negative cells derived from KhES-1 were mixed, seeded onto 96-well low-adhesion Lipidure-coated plates (NOF America Corp., White Plains, NY, USA) with REGM containing 10 μM ROCK inhibitor Y-27632 (Sigma-Aldrich) and cultured overnight to induce aggregate formation. The aggregates were then placed at the air-medium interface on polycarbonate filters (Millipore) with 0.4-μm pores for 1 week, with REGM replacement every 2 days. The resulting aggregates were fixed and serial sections were examined by immunostaining and imaged by confocal microscopy.

### γ-Glutamyltransferase (GGT) activity

KSP-positive/TRA1-60 negative cells derived from KhES-1 and primary non-neoplastic human renal proximal tubular epithelial cells (RPTEC; Lonza, Cologne, Germany) (1 × 10^6^) were homogenized in 200 μL ice-cold GGT Assay Buffer. γ**-**GGT activity colorimetric assay kit (MAK089; Sigma-Aldrich) was used according to the manufacturer’s protocol.

### Animal experiments

All mice experiments were approved by the Institutional Animal Center at Keio University School of Medicine in accordance with the National Institutes of Health Guide for the Care and Use of Laboratory Animals.

### Statistical analysis

The results are presented as the means ± S.E.M. Comparisons between groups were performed using a two-tailed Student’s t test. P < 0.05 was considered significant.

## Additional Information

**How to cite this article**: Yamaguchi, S. *et al*. Generation of kidney tubular organoids from human pluripotent stem cells. *Sci. Rep.* **6**, 38353; doi: 10.1038/srep38353 (2016).

**Publisher's note:** Springer Nature remains neutral with regard to jurisdictional claims in published maps and institutional affiliations.

## Supplementary Material

Supplementary Information
