# Supplementary material for: Generation of kidney tubular organoids from human pluripotent stem cells
Source: Sci Rep. 2016 Dec 16;6:38353. doi: 10.1038/srep38353 (PMC5159864; doi:10.1038/srep38353)
Supplement: Supplementary Information [file srep38353-s1.pdf]

Generation of kidney tubular organoids from human pluripotent stem cells

Shintaro Yamaguchi[1], Ryuji Morizane[1, 2, 3, 4], Koichiro Homma[1,5*], Toshiaki Monkawa[1, 6], Sayuri Suzuki[1], Shizuka Fujii[1], Muneaki Koda[1], Ken Hiratsuka[1], Maho Yamashita[7], Tadashi Yoshida[7], Shu Wakino[1], Koichi Hayashi[1], Junichi Sasaki[5], Shingo Hori[5] & Hiroshi Itoh[1]

1. Department of Internal Medicine, Keio University School of Medicine, 35 Shinanomachi, Shinjuku-ku, Tokyo 160-8582, Japan

2. Renal Division, Brigham and Women's Hospital, 75 Francis Street, Boston, MA 02115, USA

3. Department of Medicine, Harvard Medical School, 25 Shattuck St, Boston, MA 02115, USA

4. Harvard Stem Cell Institute, 7 Divinity Ave, Cambridge, MA 02138, USA

5. Emergency and Critical Care Medicine, Keio University School of Medicine, 35 Shinanomachi, Shinjuku-ku, Tokyo 160-8582, Japan

6. Medical Education Center, Keio University School of Medicine, 35 Shinanomachi, Shinjuku-ku, Tokyo 160-8582, Japan

7. Apheresis and Dialysis Center, Keio University School of Medicine, 35 Shinanomachi, Shinjuku-ku, Tokyo 160-8582, Japan

*Correspondence and requests for materials should be addressed to Koichiro Homma (email: homma@keio.jp)

Department of Internal Medicine, Keio University School of Medicine, 35 Shinanomachi, Shinjuku-ku, Tokyo 160-8582, Japan Tel: +81-3-3353-1211

Figure legends

Supplemental Figure 1. The expressions of nephron progenitor markers in hESCs.

a-c) Quantitative evaluation of gene expression of nephron progenitor markers (*GDNF*, *CITED1*, *HOXD11*) on day 3 and 10 of differentiation. Transcript expression levels were normalized to *GAPDH* (n =3). Values shown are the means ± SEM.

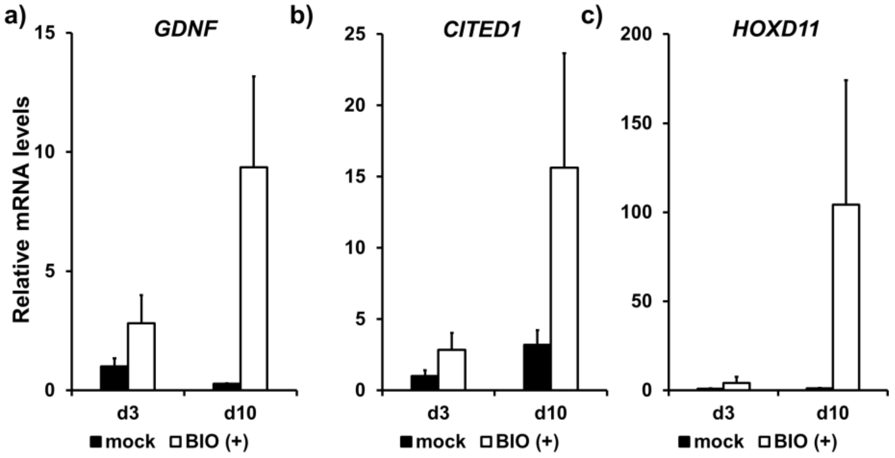

Supplemental Figure 2. Structures generated by KSP negative cells in 3D Matrigel.

KSP nagative/TRA1-60 negative cells purified by flow cytometry after 10 d of differentiation were transferred onto Matrigel and cultured for 24h. KSP negative cells formed petal-like structures which were negative for KSP and MEGALIN expression. Scale bars; 50 μm.

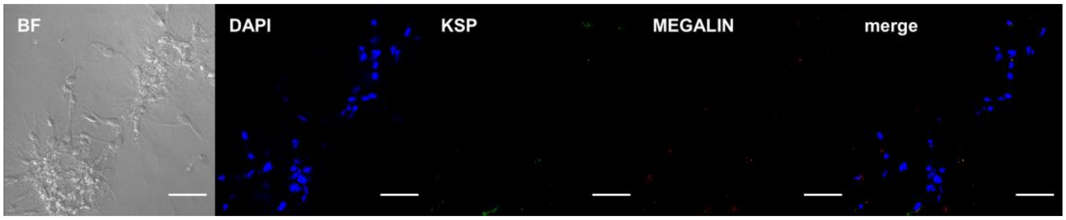

Supplemental Figure 3.

Primers for quantitative Real-Time. PCR, polymerase chain reaction.

Gene		Sequence
OCT3/4	Forward	TGA AGC TGG AGA AGG AGA AGC TG
	Reverse	GCA GAT GGT CGT TTG GCT GA
BRACHYURY	Forward	GAC AGG TAC CCA ACC CTG AGG
	Reverse	AGC ATG GAT AAA CAT GCA GGT GAG
PAX2	Forward	TGT CAG CAA AAT CCT GGG CAG
	Reverse	GTC GGG TTC TGT CGT TTG TAT T
OSR1	Forward	AGA GTC CTG GAC TGG CAG AAT C
	Reverse	AGA CCA CAG ATA TAT TCA CTC CAA AGA G
WT-1	Forward	CAC AGC ACA GGG TAC GAG AG
	Reverse	CAA GAG TCG GGG CTA CTC CA
KSP	Forward	CAT CCT TGT AGG CAC CCT GGT AG
	Reverse	TGA CAT GGT CCA GTG GGT GAA
MEGALIN	Forward	GGC CTG CTA TAA CAC CAG TCA
	Reverse	ACT CAT TGT GCA AGC ATA TCT CA
AQP 1	Forward	TAA CCC TGC TCG GTC CTT TG
	Reverse	AGT CGT AGA TGA GTA CAG CCA G
AQP 2	Forward	GCT CCG CTC CAT AGC CTT C
	Reverse	GGG TGC CAA TAC CCA AGC C
PAX6	Forward	TGGGCAGGTATTACGAGACTG
	Reverse	ACTCCCGCTTATACTGGGCTA
NKX2.5	Forward	CCAAGGACCCTAGAGCCGAA
	Reverse	ATAGGCGGGGTAGGCGTTAT
GGT	Forward	TGAGCCCAGAAGTGAGAGCAGTTG
	Reverse	ATGTCCACCAGCTCAGAGAGGGT
CD13	Forward	GACCAAAGTAAAGCGTGGAATCG
	Reverse	TCTCAGCGTCACCCGGTAG
ATP1B1	Forward	CCGGTGGCAGTTGGTTTAAGA
	Reverse	GCATCACTTGGATGGTTCCGA
SGLT1	Forward	TACCTGAGGAAGCGGTTTGGA
	Reverse	CGAGAAGATGTCTGCCGAGA
UROMODULIN	Forward	CGGCGGCTACTACGTCTAC
	Reverse	GTCTGTGCAGTACGCCAGG
SLC12A3	Forward	CCTGGGTGGAGACCTTCATTC
	Reverse	GAGCCCCAATTTACCTCTGGC
GDNF	Forward	GGCAGTGCTTCCTAGAAGAGA
	Reverse	AAGACACAACCCCGGTTTTTG
CITED1	Forward	CCTCACCTGCGAAGGAGGA
	Reverse	GGAGAGCCTATTGGAGATCCC
HOXD11	Forward	AAAAAGCGCTGTCCCTATACCA
	Reverse	TGAGGTTGAGCATCCGAGAGA
GAPDH	Forward	GTC AGT GGT GGA CCT GAC CT
	Reverse	AGG GGT CTA CAT GGC AAC TG
